# DOUBLE PROTECTION: Reaching Accord on the Ethical Conduct of Child Observational Research

**DOI:** 10.1289/ehp.117-a354

**Published:** 2009-08

**Authors:** Julia R. Barrett

**Affiliations:** **Julia R. Barrett**, MS, ELS, a Madison, Wisconsin–based science writer and editor, has written for *EHP* since 1996. She is a member of the National Association of Science Writers and the Board of Editors in the Life Sciences

From Bernardino Ramazzini’s visits to seventeenth-century craftsmen’s worksites—where he made observations that earned him the title of “father of occupational medicine”—to modern-day research leading to bans on public smoking, observational studies have improved and enhanced environmental health. Such studies involve observing people’s everyday lives, defining the characteristics of their environments, and determining whether any risks arise from activities within the context of those environments. Modern environmental observational studies, including the recently launched National Children’s Study, also measure environmental compounds and their metabolites in people’s bodies while assessing sources and routes of exposure so that the appropriate agencies can initiate reduction strategies if high exposures are seen.

Generally, an observational study is conducted without the observer intervening with research subjects to avoid undermining research goals. However, strict adherence to this practice could create significant ethical concerns, particularly when a vulnerable subject such as a child is at risk for harm. Misunderstandings and disagreements about how those concerns should be addressed in children’s observational exposure studies have forced researchers and policy makers to more precisely—and now possibly successfully—define what constitutes a scientifically rigorous and ethical study.

## Watch and Learn

Because of their behaviors, stages of development, and smaller size, children often experience a higher level of risk from environmental contaminants than adults. This was recognized in legislation such as the Food Quality Protection Act of 1996, which defined new limits for pesticide exposures and added a tenfold safety margin to previously established limits to provide better protection for children. However, such added protection may be inadequate. “Studies that have been conducted on pesticide exposure in children indicate that assuming kids are affected at tenfold-lower levels than adults is probably an underestimate and not protective enough,” says Michael Lebowitz, a retired professor of medicine and epidemiology at The University of Arizona. “We need to understand how to protect the kids, not just what to regulate.”

Additionally, pesticides are not the only contaminants to which children are exposed. Data are lacking on a large number of chemicals, including plasticizers and components of personal care products, with regard to exposure levels and the impacts they may have on children. “We don’t have the information now to be able to fully regulate,” says Lebowitz. “Sometimes we regulate on incomplete information, and sometimes we don’t regulate the right way or enough. There are so many toxic compounds that haven’t been examined sufficiently for us to know whether to ban them or regulate them.”

Furthermore, even well-regulated contaminants such as lead remain a public health concern. Decades of lead regulations have resulted in a significant decrease in the amounts found in children’s bodies, but children nevertheless remain at risk. No safe blood lead level has been established, and exposure is a continuing problem—for example, through lead paint being used on imported toys and in the manufacture of artificial turf playing fields. “There are situations where there have been exposures, and there still are exposures. The reason why is that we have an inadequacy in understanding how children come in contact with things and are actually exposed,” says Paul Lioy, a professor of environmental and occupational medicine at the University of Medicine and Dentistry, New Jersey–Robert Wood Johnson Medical School.

Groups that have opposed certain observational exposure studies, such as the Washington, DC–based nonprofit Environmental Working Group (EWG), agree that studies are needed, but their design, intended end points, and potential conflicts of interest have been stumbling blocks. “There are a lot of observational data gaps just in terms of kids’ general behavior that would extend beyond pesticide exposure to other contaminants,” says Sonya Lunder, a senior analyst at EWG. “Can we fill in some of these research gaps by observing children in less-hazardous settings?”

Moreover, long-term observational studies that involve pesticides and other contaminants may not be in child participants’ best interests, says Lunder. “What is the added benefit of observing kids for years on end as opposed to a shorter-term or cross-sectional study?” she asks. “Does the additional information gleaned in a given study outweigh the fact that we may be condoning or permitting potentially unsafe exposures to continue for years?”

Roy Fortmann, acting director of the Human Exposure and Atmospheric Sciences Division in the EPA National Exposure Research Laboratory, explains that longitudinal studies are important because they help understand variability of exposure among individuals and even within an individual’s day-to-day routine. “It is very useful to make multiple measurements of exposures over time, which might be over days, weeks, or years, to understand this variability and the factors that affect the exposures,” he says. “For many chemicals, the intra-person variability is much higher than the inter-person variability; this has been demonstrated in air pollution studies.”

## A Question of Ethics

Complexities involving the design of observational studies have sometimes led to misunderstandings, with studies and research proposals being cancelled amid charges of being unethical. One notable example is the Children’s Environmental Exposure Research Study (CHEERS), announced in 2004, which was funded in part by the U.S. Environmental Protection Agency (EPA). The study was designed to characterize children’s exposure to pesticides and other chemicals over a two-year period through questionnaires, biomonitoring, and analysis of samples collected in their homes. Children would not have been deliberately exposed to chemicals, nor were families required to use pesticides to qualify for the study. However, Lunder says this was not clear from the initial study materials, and critics worried that prospective participants, especially low-income families, might initiate or increase pesticide use in order to receive the financial compensation offered by the study.

Opponents of CHEERS, including EWG and California Senator Barbara Boxer, who heads the Senate Committee on Environment and Public Works, objected to the study due to “the fundamentally unethical nature of the study design, which proposed to stand by and simply observe what the study acknowledged to be high pesticide exposures to infants and small children,” as EWG senior vice president Richard Wiles wrote in a January 2005 letter to then–EPA Administrator Stephen L. Johnson. Critics also cited funding from the American Chemistry Council, a chemical industry trade association, and a concentration of poor, minority families in the study as ethical concerns.

According to the opponents, children could be endangered by a plan to simply observe them and to not intervene regardless of what was seen. “Families were being selected because they were likely to engage in risky behaviors, yet they were not informed from the outset that this was the reason for the study,” says Lunder. “It also appeared at the time that there wasn’t any plan to intervene if pesticide storage practices or biological measurements indicated risk to children. Observational studies cannot observe high-risk behaviors without a plan to intervene when exposures are potentially unsafe.”

According to Lioy, these misrepresentations arose from misunderstandings on both sides. “I think the opposition was based upon the fact that people didn’t quite understand what the EPA was doing in its studies,” he says. He suggests that “things were not as clearly defined as they should have been,” and opponents may have inferred from the wording of the study’s Request for Proposals that exposures would be deliberate.

On the basis of such objections, the study was halted in November 2004 pending an internal review of the study. The review, conducted by the EPA National Exposure Research Laboratory, found that the study complied with federal regulations and had the approval of four independent institutional review boards. Additionally, the reviewers noted that the study required intervention and mitigation should improper pesticide use or high exposures be detected and also concluded that families of low socioeconomic status had not been targeted. Nevertheless, the EPA cancelled the study on 8 April 2005, citing problems created by misrepresentations of the study and the ensuing controversy.

CHEERS illustrates the need for researchers to explain clearly and precisely how they are designing observational studies to demonstrate that deliberate exposure is not involved. “In observational studies, we don’t increase a child’s exposure due to participation in the study. We only observe and measure what is already going on, and we do it in [children’s] normal environment as they go about their normal activities,” says Fortmann. Lioy adds, “Until we prevent the exposures from happening to begin with, we need to complete observational studies to ensure the exposures are not excessive.”

## Tailoring Guidance to Fit

The CHEERS episode also emphasizes a critical flaw in human study regulations: none of them pertain specifically to observational studies. The guidelines in use are an inexact fit, with noticeable gaps and strained boundaries. Consequently, after the cancellation of CHEERS, the EPA, policy makers, and a cross-section of experts in ethics and exposure research, together and separately, scrutinized the adequacy of existing safeguards for observational studies.

The primary set of rules, which have applied to almost all human research studies conducted or funded by the federal government since 1991 (with exemptions for certain types of studies such as educational tests), is the Federal Policy for the Protection of Human Subjects (45 CFR § 46). The Common Rule, as it is often called, draws on earlier literature that outlines three principles of ethical research: respect for individuals’ autonomy, which includes the idea of informed consent; beneficence, which emphasizes maximizing benefits and minimizing risks to individuals and society; and justice, which requires fair and balanced treatment of all groups and individuals.

The Common Rule defines all components of a human research study. It spells out criteria for institutional review board membership, authority, and protocols, as well as elements required for informed consent. The EPA has also promulgated regulations beyond the Common Rule that provide additional protections for children, pregnant women, and nursing mothers. However, the Common Rule was written with medical research studies in mind.

Closer to the goal but still falling short is the EPA’s Protections for Subjects in Human Research Rule, which went into effect 7 April 2006 and almost immediately became the subject of a lawsuit (a settlement is currently being discussed). However, this rule covers intentional exposure research, studies in which subjects are deliberately given a specific amount of a substance—a characteristic that does not apply in observational exposure studies.

“The same basic regulations and guidelines for research with human subjects apply to observational and interventional studies. [But] there are no regulations or guidelines that deal specifically with observational research,” says David B. Resnik, an NIEHS bioethicist. To overcome this shortcoming, the EPA sponsored an expert panel workshop in late 2006 to specifically consider observational exposure studies, placing emphasis on key elements such as design, protection of vulnerable groups, and clear communication among researchers, participants, the public, and other stakeholders. After expert and public reviews, the EPA released the final report from this meeting, *Scientific and Ethical Approaches for Observational Exposure Studies* (SEAOES), in May 2008.

As recently as September 2008, however, the EPA was compelled to withdraw Requests for Proposals on two new observational studies. At Senator Boxer’s request, the Committee on Environment and Public Works pointedly questioned the EPA about these proposals, after which the agency decided to cancel them pending further incorporation of the ethical considerations discussed in SEAOES into agency policy.

## Toward Better Communication

In an effort to promote dialog and to resolve lingering misunderstandings, a group of senior exposure researchers that included Lebowitz and Lioy collaborated on “The Necessity of Observing Children’s Exposure to Contaminants in Their Real-World Environmental Settings,” a white paper posted on the International Society of Exposure Science website in December 2008. (A slightly revised version of the paper was later posted on the website of the International Society for Environmental Epidemiology.) The authors emphasized the necessity of observational studies and bluntly stated that not pursuing this knowledge is in itself unethical.

Lebowitz says observational studies are essential to know what is happening in the real world so better protections can be formed. “We think that a lot of the increased effects in children compared with adults due to various contaminants have probably been underestimated,” he says. “So, these studies will probably lead to even more rigorous statutes, rules, and regulations to protect children.”

The white paper also called for better communication between all stakeholders, including members of Congress. Clear and effective communication must include investigators and subjects, as well as the community and public, according to Resnik (who was not a coauthor). “It is important that the subjects, community, and public understand the nature of the research and why it is important,” he says. “They need to understand that children are being observed, not experimentally manipulated. Investigators need to communicate this clearly in the way they write their protocols, consent forms, advertisements, and other study documents.”

Lebowitz believes stakeholders are finding accord. “Congress, both sides, has been much more favorably interested in this research than the Bush administration itself, which didn’t even want the National Children’s Study to go forth,” he says. “That—plus some proactive, positive steps that a number of us have taken to stimulate discussion and stimulate changes within the EPA—have, I think, been beneficial.”

According to the Committee on Environment and Public Works, this optimism is well-founded. An updated ethical guidance document that consolidates lessons learned from CHEERS and other studies is currently under review.

“I think the path is becoming clearer because both the EPA and Congress are talking,” Lioy says. “There was just a lot of miscommunication and ambiguity at one point in time. Hopefully, this will be resolved, because the country needs these kinds of studies.”

## Figures and Tables

**Figure:**
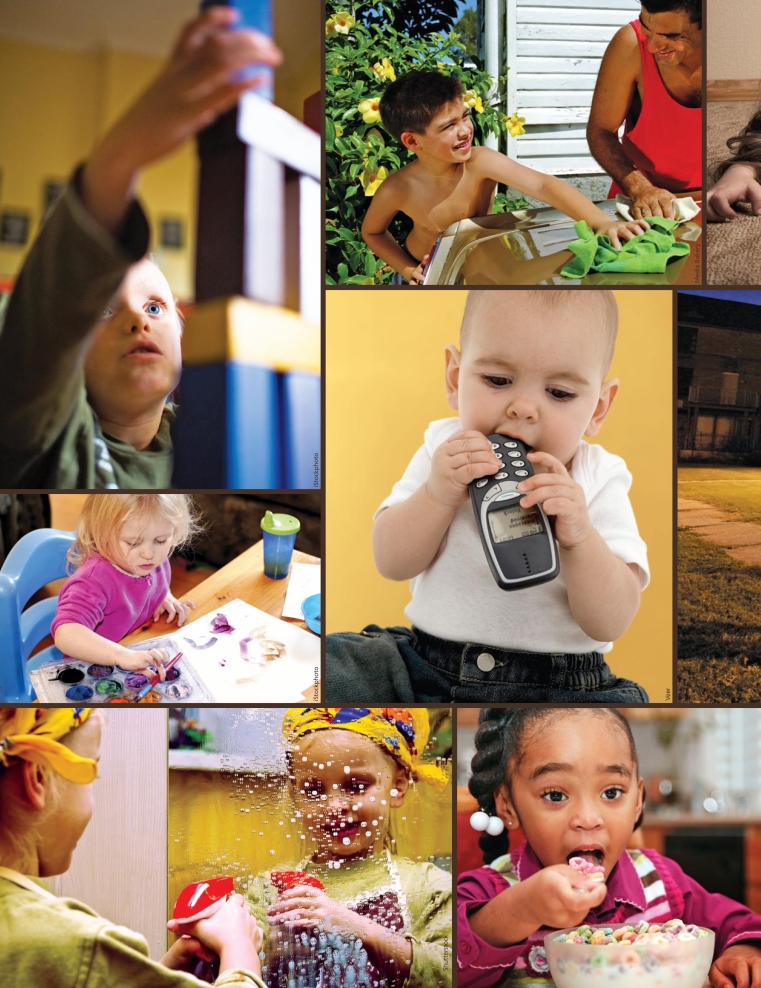
Children are often at greater risk than adults for adverse effects from environmental agents. But without a better understanding of when, where, how, and why children’s exposures occur, reliable protection is impossible.

